# Program managers’ perspectives on using knowledge to support population health management initiatives in their development towards health and wellbeing systems: a qualitative study

**DOI:** 10.1186/s12961-023-01057-8

**Published:** 2023-10-17

**Authors:** N. J. E. van Vooren, H. W. Drewes, E. de Weger, I. M. B. Bongers, C. A. Baan

**Affiliations:** 1 Department of Quality of Care and Health Economics, Centre for Nutrition, Prevention and Health Services, National Institute for Public Health and the Environment (RIVM), P.O. Box 1, 3720 BA Bilthoven, The Netherlands; 2 Tilburg School of Social and Behavioural Sciences, Tranzo, Tilburg University, PO Box 90153, 5000 LE Tilburg, The Netherlands; 3 Siza, PO Box 532, 6800 AM Arnhem, The Netherlands; 4 Vrije Universiteit Amsterdam, Athena Instituut, de Boelelaan 1085, 1081 HV Amsterdam, The Netherlands; 5 Mental Health Care Institute Eindhoven, de Kempen, PO Box 909, 5600 AX Eindhoven, The Netherlands

**Keywords:** Knowledge mobilization, Health systems transformation, Population health management, Learning

## Abstract

**Background:**

Population health management (PHM) initiatives are more frequently implemented as a means to tackle the growing pressure on healthcare systems in Western countries. These initiatives aim to transform healthcare systems into sustainable health and wellbeing systems. International studies have already identified guiding principles to aid this development. However, translating this knowledge to action remains a challenge. To help address this challenge, the study aims to identify program managers’ experiences and their expectations as to the use of this knowledge to support the development process of PHM initiatives.

**Methods:**

Semi-structured interviews were held with program managers of ten Dutch PHM initiatives. These Dutch PHM initiatives were all part of a reflexive evaluation study and were selected on the basis of their variety in focus and involved stakeholders. Program managers were asked about their experiences with, and expectations towards, knowledge use to support the development of their initiative. The interviews with the program managers were coded and clustered thematically.

**Results:**

Three lessons for knowledge use for the development of PHM initiatives were identified: (1) being able to use knowledge regarding the complexity of PHM development requires (external) expertise regarding PHM development and knowledge about the local situation regarding these themes; (2) the dissemination of knowledge about strategies for PHM development requires better guidance for action, by providing more practical examples of actions and consequences; (3) a collective learning process within the PHM initiative is needed to support knowledge being successfully used for action.

**Conclusions:**

Disseminating and using knowledge to aid PHM initiatives is complex due to the complexity of the PHM development itself, and the different contextual factors affecting knowledge use in this development. The findings in this study suggest that for empirical knowledge to support PHM development, tailoring knowledge to only program managers’ use might be insufficient to support the initiatives’ development, as urgency for change amongst the other involved stakeholders is needed to translate knowledge to action. Therefore, including more partners of the initiatives in knowledge dissemination and mobilization processes is advised.

**Supplementary Information:**

The online version contains supplementary material available at 10.1186/s12961-023-01057-8.

## Introduction

Health systems in Western countries are under growing pressure to provide appropriate, high-quality and affordable healthcare for their populations. Due to technological developments and a rising demand for healthcare, healthcare costs are increasing [1]. In addition, there is a growing shortage of healthcare staff, and demands for health and care are changing among the ageing populations [2, 3]. As a way to address this growing pressure, multiple population health management (PHM) initiatives have been implemented. These initiatives aim to transform from healthcare systems towards sustainable and integrated health and wellbeing systems. This means that they will be integrating and reorganizing services across multiple sectors, including those of healthcare, public health, social care and community services [4]. They will be, for instance, responsible for providing joined-up care for a certain population, which includes overcoming sector-specific boundaries [5, 6]. Examples of these initiatives are Gesundes Kinzigtal in Germany [7–9], Accountable Care Organizations in the United States [10], the Ontario Health Teams in Canada [5], the Integrated Care Systems in the United Kingdom [6] and the Population Health Management (PHM) sites in the Netherlands [11]. The transformation process of these initiatives can be described as a cyclical and non-linear learning process with feedback loops and with interaction between the initiatives and the larger systems and structures they are in [12]. While attempting new ways of organizing health and care services, these PHM initiatives have to overcome long-standing vested interests, unsuitable system structures (e.g. accountability and finance structures focused on the separate sectors) and differences in power, meaning and value among the partners of the initiatives [11, 13–16].

With the aim of supporting this development, international studies have identified empirical knowledge, which is based on structured data gathering, regarding different strategies that are of importance for this transformation [13, 14, 17–19]. In addition, more in-depth knowledge has been developed as to the role of context in the workings of these strategies [11, 20]. However, this knowledge about strategies alone is not enough to support the transformation. For decades, the existence of a knowledge–action gap has been recognized as one of the most important challenges for public health [21–23].

There is a growing understanding of how to address this knowledge–action gap. Multiple models have been introduced that suggest facilitating and limiting factors for knowledge dissemination and mobilization. The differences between these models were categorized by Best and Holmes [24] in three ‘generations of thinking’ about knowledge dissemination and mobilization. These vary between 1) a linear model, 2) a relations model, or 3) including a complex systems perspective [19, 24, 25]. The more linear models pursue knowledge as a product which can be exchanged from research producer to research user.  Whereas a relations model such as that of Graham et al. [25] describes knowledge translation as a cyclical process of linking knowledge creation and action. Best and Holmes [24] further discuss the importance of a systems model for linking knowledge to action, where they recognize that diffusion and dissemination of knowledge is influenced and shaped by the contextual factors of the system, among which are the partners’ various perspectives, priorities and expectations. Despite these growing insights, knowledge dissemination and mobilization in the field of health system transformation is still found to be difficult due to the complexity of the different stakeholders' perspectives and other contextual factors that affect the use of knowledge [21, 24].

In the Netherlands, there are more than 118 regional initiatives which work across the sectors of healthcare, wellbeing and prevention [26]. Since 2014, the Dutch National Institute for Public Health and the Environment (RIVM) has been involved in knowledge creation on strategies for the development process towards sustainable health and wellbeing systems  [11, 27]. To enable the Dutch initiatives to act on empirically derived knowledge, and to also take into account the complexity of context, the authors identified eight guiding principles for PHM development including information about what strategies worked, why, and in what context (See Textbox 1) [11]. While this process started off from a linear knowledge dissemination perspective, the authors became aware that for usefully disseminating this knowledge into the complex developments of the Dutch initiatives, better connection with the contexts of these initiatives was evident. This is more in line with the relationship models and the complex systems perspective on knowledge dissemination. This study therefore aimed to explore how to successfully use empirical knowledge for the developmental processes of Dutch PHM initiatives. Within these initiatives, program managers were expected to play an overarching role in knowledge use, in the literature this is also called ‘knowledge broker’ [28], since they were the ones who were assigned with facilitating the development of the PHM initiatives. To learn more about how to apply the knowledge of the guiding principles for PHM development, this study therefore focused on the experiences and expectations of the program managers of the initiatives.

The main research questions were:

What are program managers’ experiences and expectations as to the use of empirical knowledge to further support the development of PHM initiatives aimed at transforming into health and wellbeing systems?

- How are program managers currently using knowledge for PHM development?
- How can the knowledge of guiding principles for PHM development be used to successfully further develop PHM initiatives?

Textbox 1. Knowledge base used [11] Eight guiding principles for developing towards a health and wellbeing system

1. Create and maintain commitment between organizations while working towards a health and wellbeing system.

2. Achieve mutual understanding of norms, values and roles, and create trust.

3. Define preconditions for accountability to be able to share both successes and risks.

4. Ensure regional agreements are underpinned by political support to influence policy development.

5. Make sure that the financial incentives align with overarching system goals.

6. Ensure a learning cycle by developing a data and knowledge infrastructure on both the organizational and the regional level.

7. Enable community involvement and gain insight as to communities’ needs.

8. Provide suitable stakeholder representation and suitable leadership to promote development towards a health and wellbeing system.

## Method

### Setting

This study is part of the reflexive evaluation ‘the right care in the right place’ from the Dutch National Institute for Public Health and the Environment [29]. The aim of the reflexive evaluation is to learn what PHM initiatives need in their development towards health and wellbeing systems, and to aid and inspire this development. A total of ten Dutch PHM initiatives were selected to take part in the reflexive evaluation study. These PHM initiatives were selected as they varied in scope (rural/urban, regional scope or city scope), stakeholders, and developmental phase and therefore could provide insights related to a broad scope of experiences [29]. As part of this reflexive evaluation, this study focused specifically on the use of knowledge in these ten selected PHM initiatives.

### Data collection

The RIVM researchers had already gone through a knowledge-creation cycle to identify guiding principles for the development of PHM initiatives (see Textbox 1, based on van Vooren et al. [11]). To learn how this knowledge could aid practice, ten semi-structured interviews were performed among the program managers of the PHM initiatives in the reflexive evaluation study.

First, one of the program managers was interviewed in June 2021 about the experiences with a facilitated reflection process in the initiative (performed in May 2021). Here the knowledge of the guiding principles was used to facilitate the reflection on the collaboration process. Being part of the reflexive evaluation, the program manager had asked for help with facilitation of their reflection process. Afterwards the program manager was interviewed about (1) the use of knowledge prior to this reflection process, and (2) the experiences with using the knowledge of the guiding principles in the facilitated reflection process.

Between June and September 2022, nine additional interviews were organized with the program managers of the other nine initiatives involved in the reflexive monitor about their experiences with using knowledge, and experiences and expectations of using knowledge like the guiding principles (from Textbox 1) for their development process. These program managers were invited by email and all of them agreed to participate in the study. In one region a second program manager from the initiative joined the interview as well, and in another region a knowledge manager with an overarching perspective on the initiative also participated in the interview together with the program manager. The interview questions for this study were focussed on (1) the use of knowledge by program managers in the PHM initiatives, and (2) their experiences and expectations in using knowledge like the guiding principles for the developmental process of the PHM initiatives, and what was needed to be able to use this knowledge (see Additional file 1 for the full interview guide). The context of the facilitated reflection process in 2021, was used as an example in these interviews to show how the knowledge of the guiding principles could be used in practice (see the interview guide in Additional file 1 for the examples that were given for showing, and scoring on behalf of, the knowledge base of the guiding principles).

## Analysis

All interviewees (*N* = 12) provided consent for the study and use of the data. The study and the informed consent forms were approved by the Ethical Review Board of Tilburg University (RP 252). The interviews with the program managers of the PHM initiatives were transcribed literally.

The transcripts of the interviews with the nine PHM initiatives were coded in MaxQDA2022. The interviews were analysed in line with the steps of thematic analysis described by Braun and Clarke (2006). The coding was both deductive and inductive. The coding process started with initial deductive codes based on the interview questions, which included (1) the current use of knowledge and (2) the needs for using the guiding principles. Throughout the coding process, new (sub)codes were added inductively. One of the researchers (NvV) coded the interviews as first coder, and the other (EdW) coded the interviews as second coder. The codes were clustered into themes by NvV, and these were checked by EdW.

As the interviews were performed in Dutch, the quotes that are used in this article were translated from Dutch to English.

## Results

This results section will first describe the current use of knowledge by program managers for PHM development, and their perception on the usefulness of the themes of the eight guiding principles. Then, three lessons about what is needed for using empirical knowledge like the guiding principles will be described.

### Current use of knowledge for PHM development

The results of the interviews showed that program managers used different sources of knowledge to aid the initiatives’ development process. These were, amongst others, different models and reports about transition processes and building blocks for collaboration, inspirational videos about culture change, and program managers participated in training programs. Program managers said to check this knowledge on usefulness for their own local context. For example, one program manager selects information on relevance before communicating it with the rest of the initiative partners. Another program manager said to actively search for knowledge only when a question arose. As such, for this program manager, proactive knowledge dissemination would not be suitable; instead, reactive knowledge translation based on regional questions might be a better fit.

*“Yes, I think that is also very much the quest, it seems to me, of every coordinator [...] you set off, so to speak, you come across certain things. And that's actually the […] moment that you [require] a forum where you can then find information about the point that you encounter. Uhm and then what doesn’t help me is a report of 60 pages. But, what would help then, is maybe [..] that you know which question box you can go to and ask that question and get a tailor-made answer. Or a half-hour conversation with someone who says, […] think about this for a moment, think about that. Or then the advice of, go and read that report of 30 pages, because you'll find it in there” (I9_program manager).*

In addition, one program manager mentioned that knowledge is only one of the factors that can aid the development process and thus questions whether regular reflection with a structured list of themes for the development is necessary. According to this program manager, choosing whether to use empirical knowledge is dependent on the context. Sometimes resorting to other strategies, such as having a mutual conversation to build trust, might be a better fit to aid the development process.

*“There is a huge toolbox that you can draw from and where one time you take out one tool, namely the hard data, the next time you focus on having more in-depth one-on-one conversations. And the next time you choose for a group discussion” (I2_program manager).*

### Reflection on the usefulness of the guiding principles for PHM development

When asking specifically about the use of the guiding principles from Textbox 1 for the development process, the program managers appreciated the broad representation of themes within the guiding principles and recognized the themes from their own practice. Two program managers explicitly mentioned that these principles showed them themes that they had not focused on yet (e.g. accountability). One program manager mentioned the need to have a broad perspective on the development of the regional initiative:

*“[...] because I recognize this [the themes of the guiding principles] very much, and actually you have to bear all these in mind and be able to steer on all these, if you want to make the movement in, in its totality. [...] And I see a lot of collaborations, because I’m aware of a lot more of them, of course, and I’m involved in a number of others [initiatives] myself. Those are [focused] on such small pieces. And they do fine work on that small piece. But that’s not about the whole” (I2_program manager).*

The program manager who experienced the use of the guiding principles within the facilitated reflection process mentioned that by using these guiding principles all relevant points for discussion were addressed. In addition, according to this program manager, there had previously been regular reflection moments that focused on the practical results within the initiative (for example monitoring of interventions). However, reflection on process and governance, like in this facilitated reflection process, was less structured and was mainly conducted during decision-making stages. Also, going through the facilitated reflection process had been the first time that the initiatives’ partners discussed a broad and long-term focus of their initiative, as previously they focussed on subtracted themes within the larger process. The importance of reflecting on the collective development process in addition to using data about the progress of interventions was mentioned in the interviews with the program managers of the other PHM initiatives as well.

### Lessons learned on using of the guiding principles in practice

Firstly, a need for (external) expertise to be able to use knowledge was identified. For example, due to the broad representation of themes within the guiding principles, program managers mentioned that expertise was needed within the regional initiatives to know how to properly use these guiding principles to aid their developmental process. This included both expertise about the meaning of each principle as well as expertise about the local situation (e.g. understanding the financial incentives of  the regional partners). This expertise was integrated in some initiatives, as some program managers were part of a larger supporting organization with experience in regional collaboration. In one initiative, a knowledge broker had been hired to aid the program manager in the use of knowledge in the PHM process, as the program manager felt too involved in the PHM system to be able to invest in, and reflect on the learning process properly. In other initiatives, researchers and PhD students were involved to provide expertise on different subjects regarding the development of the initiatives. Two program managers also appreciated the exchange of knowledge between the initiatives themselves. Other initiatives had asked consultancies to facilitate the reflection on their developmental process. In addition, the program manager who experienced the facilitated reflection proces valued  the support of an independent researcher. According to the program manager, regional partners had mentioned that by having individual evaluation interviews with the researcher, they felt seen and valued for their input. In addition, the program manager valued having research-based insights about the development of the initiative, which were experienced as more grounded than the program managers’ own previous assumptions.

Secondly, the broad reflection on the PHM initiatives based on the guiding principles was valued, but the dissemination of this knowledge required better guidance for action. As a result, program managers came up with several suggestions for disseminating the knowledge for better use. One program manager valued action-oriented and short descriptions of the principles as a basis for looking for further information when needed. Another program manager found that the language of the guiding principles currently did not fit their PHM initiatives’ vision. Program managers of another initiative reacted to the idea of using scoring methods, such as the radar chart that was used in the reflection process in 2021 (see an example of a radar chart in Additional file 1: Fig. S3), as a way to visualize the situation in the regional initiative and provide opportunities to improve. Although, how to act based on this knowledge would then require further guidance. One program manager, for instance, reflected on the difficulty of choosing which principle to focus on after scoring them (should you start with the lowest score or the highest?). The program managers’ colleague added that this scoring would need to be ‘experienced’ by the partners for them to act on it, meaning that they would have to understand the consequences of choosing to act (or not) on the basis of the scoring. Additionally, also in the facilitated reflection process in 2021 more concrete examples for action based on the results of the reflection process would have been valued, according to the program manager.

Thirdly, some program managers mentioned the need for having a collective learning process in the initiative for this knowledge to be used in practice. Two program managers mentioned that first a basis of trust was needed before reflection based on themes such as the guiding principles would be useful. This requires openness, for example regarding the difficulties that organizations experience when balancing their own interests with those of the collaborative.

“[…] and being able to talk to each other about what that means for you as a person uhm and how you do that with your organization, and actually having the guts to be very open and honest about it. Look, it’s also about […] do you dare to share your own budgets with each other? Do you dare to give each other real insight into how it operates behind the scenes?” (I3_program manager).

Some program managers had experience in facilitating a structural learning process, for example, by having quarterly or annual reflection meetings or brainstorm sessions. Other program managers had not (yet) invested in a structural collective learning process among the regional partners (director or manager level). According to the program managers, the sense of urgency of the regional partners for collective learning influenced the investment in this learning process. For example, one program manager mentioned that due to the early developmental phase they were in, partners were more keen to look forward in their development than to reflect. Other program managers mentioned the board members’ preference to focus on doing rather than on reflecting, with one of the reasons for this being the time needed for other daily priorities. Furthermore, the relationship between using a knowledge base for reflection, and the existence of a collective learning environment for this reflection appeared to be bidirectional. For example, some program managers expected that having a reflection moment based on themes such as the guiding principles could be useful in boosting the energy among the partners for the transformation process they are going through. In addition, the program manager who had experienced the facilitated reflection process reflected on the two meetings in which the involved partners reflected together on the scoring of the initiative as being helpful in getting the conversation going for the collective process.

## Discussion

This study aimed to learn about the key insights for using knowledge in practice to aid the development of PHM initiatives. On the basis of the experiences and expectations of the interviewed program managers we learned that knowledge like the guiding principles was used and valued as one of the means to aid the developmental process of PHM initiatives. Three lessons on knowledge use for the development of PHM initiatives were identified: (1) being able to use knowledge on the complexity of PHM development requires (external) expertise about themes regarding PHM development and knowledge about the local situation regarding these themes; (2) the dissemination of knowledge about strategies for PHM development requires better guidance for action by providing more practical examples of actions and consequences; and (3) results showed that a collective learning process within the PHM initiative is needed to support knowledge being successfully translated into action. By using knowledge about the guiding principles as an example, this study enriches the current literature base on knowledge dissemination and mobilization with more detailed information on knowledge use in the context of PHM initiative development.

As described in the introduction and also found in the results, knowledge dissemination and mobilization regarding the development of these PHM initiatives is difficult on multiple levels. First of all, we learned that knowledge about PHM development itself is complex. PHM initiatives are seen as complex adaptive systems, influenced by many different factors in their developmental process [17]. This complexity has been taken into account in the knowledge creation process that was used in this study [11]. However, the results show that using this complex information in practice proves difficult and requires expertise. To deal with this complexity, the findings in our study and in the literature suggest the need for insight into more concrete actions or insight into consequences [21]. One of the methods aimed at providing more actionable insights, including the role of context, is the realist evaluation approach. This approach was also at the base of the guiding principles in Textbox 1, however, due to the large spectrum of detailed information regarding strategies, contexts, mechanisms and outcomes, it was not discussed in such detail during the interviews. Finding a balance between the understanding of complexity and providing detailed actionable insights still proves difficult [24, 30].

According to our findings, adding to the difficulty of using knowledge about PHM development are the different contextual factors that influence the use of knowledge for PHM development. For example, the expertise of program managers, the trust among partners, the urgency for change, and the existence of a learning environment in the PHM initiatives. This is in line with the increasing focus on a systems perspective on knowledge dissemination and mobilization, which includes the recognition that dissemination processes are embedded in structures and interactions across stakeholders, and that this requires a shared learning process [24, 31]. The importance of understanding the role of ‘the system’ in knowledge use can be described by the example of the role of the program managers in the PHM initiatives in our study. We found that most of the program managers we interviewed, as expected, took up roles that are related to tasks of knowledge brokers [28]. Knowledge brokers are seen as the human component of strategies for knowledge translation, and perform tasks such as the linkage of relevant stakeholders, supporting communication, and facilitating change [28]. Despite program managers taking these roles, they argued that for actual use of, and change driven by knowledge, they need the urgency for change from the partners, that is, ‘the system’, within their initiative. We therefore consider that only tailoring knowledge to program managers might not be sufficient and it might require more facilitation of the collective conversation (as was done in the facilitated reflection process) to improve the learning process. However, this study only included the perspective of program managers, and limited insights have been retrieved on the effects of the facilitated reflection process. Therefore, further research that includes the experiences with knowledge use by other partners from the PHM initiatives is needed.

When learning about knowledge dissemination and mobilization, it is important to note that this study used the approach of ‘end-of-grant’ knowledge translation [32]. The actual use in practice was considered after the knowledge base had been created. In contrast to this we see that the use of 'integrated knowledge translation' is increasingly suggested, meaning that stakeholders are already involved in knowledge creation from the start of the process [33]. This includes a paradigm shift from scientist-driven research to more collective-problem-based research [33]. The importance of including the perspective of the users in knowledge mobilization is shown by our study as well. While the guiding principles were created with the idea of providing action-oriented knowledge for practice, current study shows that program managers still would experience difficulty in applying this knowledge in their context. Further research on understanding knowledge use for PHM initiative development should therefore aim at an integrated knowledge translation process, for example, by using participatory action research or reflexive evaluations, which could also aid in learning about the role of the system (rather than the program manager) in knowledge use. As the current study examined the use of knowledge only during first reflection steps, examining the use of knowledge for a longer period of time will be valuable to learn about its use for decision-making and actual change within the PHM initiatives.

## Conclusions

The results of this study show that, according to program managers, empirical knowledge can be used as one of the means to aid the developmental process of PHM initiatives. This translation is however complex due to the complexity of understanding PHM development itself, as well as the different contextual factors affecting knowledge use for PHM development. Lessons for using knowledge to aid the developmental process suggest investing in (external) expertise for knowledge use and providing actionable principles for PHM development. Program managers also reflected on the need for a collective learning environment as a base for knowledge use for PHM development, as they needed their regional partners to have a sense of urgency to use knowledge for their development. Therefore, including more regional partners in the knowledge dissemination process instead of only program managers is advised for future research.

### Supplementary Information

**Additional file 1: Figure S1.** Example of systems’ transformation process and the different phases of this transformation (retrieved from the Rippel Foundation, 2020 at: Stewards' Pathway - ReThink Health). **Figure S2.** Visualization of the guiding principles for PHM development. **Figure S3.** Example of a radar chart based on the guiding principles for reflecting upon the collaboration process.

## Data Availability

The datasets analysed during the current study are not publicly available but are available from the corresponding author on reasonable request. Any templates used for data collection and analysis are available from the corresponding author on reasonable request.

## Abbreviations

PHM: Population health management
RIVM: National Institute for Public Health and the Environment (Dutch: Rijksinstituut voor Volksgezondheid en Milieu)
