# Supplementary material for: Program managers’ perspectives on using knowledge to support population health management initiatives in their development towards health and wellbeing systems: a qualitative study
Source: Health Res Policy Syst. 2023 Oct 17;21:106. doi: 10.1186/s12961-023-01057-8 (PMC10583399; doi:10.1186/s12961-023-01057-8)
Supplement: Supplementary file 1 — Additional file 1: Figure S1. Example of systems’ transformation process and the different phases of this transformation (retrieved from the Rippel Foundation, 2020 at: Stewards' Pathway - ReThink Health). Figure S2. Visualization of the guiding principles for PHM development. Figure S3. Example of a radar chart based on the guiding principles for reflecting upon the collaboration process. [file 12961_2023_1057_MOESM1_ESM.docx]

# **Appendix - Interview guide**

**Part 1: Experiences with learning capacity/joined learning and reflecting for the successful transformation of PHM/regional systems**

1. **See Figure 1. The transformation that we’re talking about today takes a long time and consist of multiple phases. Do you recognise these phases within your own system?**

- *Yes/no, why? Could you provide an example?*
- *Where do you think your own systems are within these phases? [Provide explanation of the phases]*
- *What support/resources/strategies does your system need during this phase? Why?*

1. **Do you have any strategies or ways of working of learning and reflecting on a regional level regarding what steps and strategies you can, should and will take to successfully transform the system? Could you explain?**

- *Which strategies/actions/resources do you apply? Why?*
- *Which themes are being addressed, how, by whom? And how do you decide these?*
- *Do you actually learn and reflect on a system/regional level? Or is the learning and reflecting still largely taking place on an intervention-level, e.g. stoplight-methods to adjust individual interventions)?*
- *What is going well regarding the joined learning & reflecting and what isn’t working well? What are the enablers and barriers?*
- *What knowledge do you need/is missing, to make the right choices to further the transformation successfully?*
- *What do you need to successfully use this knowledge? Why?*
- *Can you provide an example of a situation where the joined learning & reflecting was improved? Which strategies, resources did you use? What effect did this have, and why?*
- *Are there situations where the joined learning and reflecting did not improve; was not successful? What went wrong, why?*

**Part 2: Knowledge translation**

1. [Explaining the background to the guiding principles for PHM development (Figure 2), and the use of this knowledge within the case study (Figure 3)]

Could this knowledge play a role in the development process of your PHM initiative?

- Why/ why not? What (other) knowledge is required?
- What is needed to be able to use this knowledge for PHM development?
- How could this knowledge be translated? In what form?


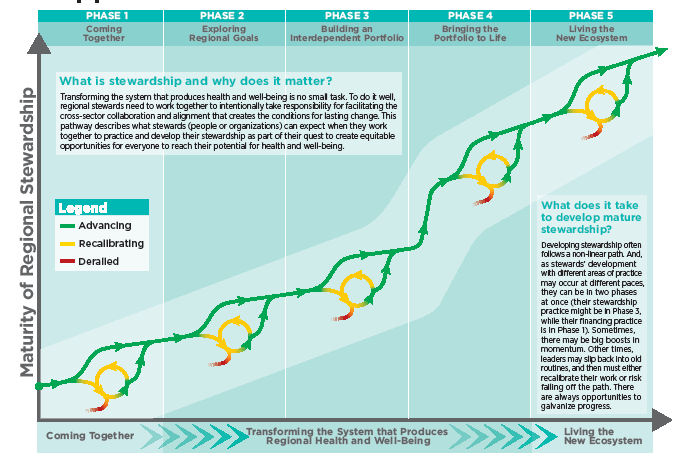


**Figure S1 *– Example of systems’ transformation process and the different phases of this transformation (Retrieved from the Rippel Foundation, 2020 at:*** [Stewards' Pathway - ReThink Health](https://rethinkhealth.org/stewards-pathway/#2)***).***


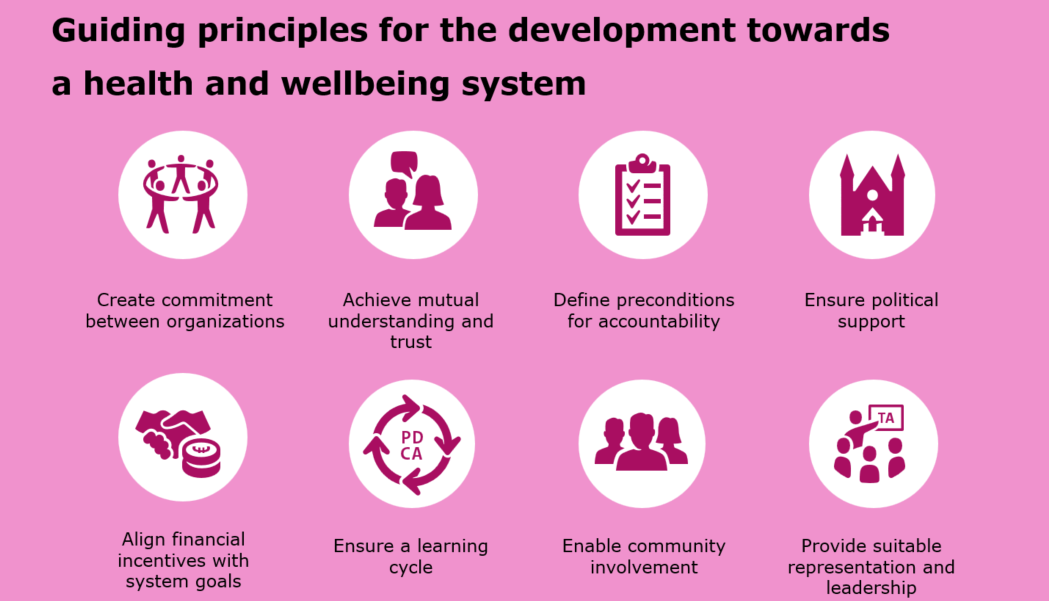


**Figure S2 – Visualization of the guiding principles for PHM development**

**Figure S3: Example of a radar chart based on the guiding principles for reflecting upon the collaboration process**
